# Independent predictors of tuberculosis mortality in a high HIV prevalence setting: a retrospective cohort study

**DOI:** 10.1186/s12981-015-0076-5

**Published:** 2015-10-06

**Authors:** Dominique J. Pepper, Michael Schomaker, Robert J. Wilkinson, Virginia de Azevedo, Gary Maartens

**Affiliations:** Department of Medicine, University of Cape Town, Anzio Road, Cape Town, 7925 South Africa; Critical Care Medicine Department, National Institutes of Health, 10 Center Drive, Bethesda, USA; Centre for Infectious Disease Epidemiology and Research, University of Cape Town, Anzio Road, Cape Town, 7925 South Africa; Clinical Infectious Diseases Research Initiative, Institute of Infectious Diseases and Molecular Medicine, University of Cape Town, Cape Town, South Africa; Department of Medicine, Imperial College, London, W2 1PG UK; City Health, Cape Town, South Africa; Division of Pharmacology, Groote Schuur Hospital, Anzio Road, Cape Town, 7925 South Africa

**Keywords:** Tuberculosis, HIV, Mortality, Predictors, Multivariate analysis

## Abstract

**Background:**

Identifying those at increased risk of death during TB treatment is a priority in resource-constrained settings. We performed this study to determine predictors of mortality during TB treatment.

**Methods:**

We performed a retrospective analysis of a TB surveillance population in a high HIV prevalence area that was recorded in ETR.net (Electronic Tuberculosis Register). Adult TB cases initiated TB treatment from 2007 through 2009 in Khayelitsha, South Africa. Cox proportional hazards models were used to identify risk factors for death (after multiple imputations for missing data). Model selection was performed using Akaike’s Information Criterion to obtain the most relevant predictors of death.

**Results:**

Of 16,209 adult TB cases, 851 (5.3 %) died during TB treatment. In all TB cases, advancing age, co-infection with HIV, a prior history of TB and the presence of both pulmonary and extra-pulmonary TB were independently associated with an increasing hazard of death. In HIV-infected TB cases, advancing age and female gender were independently associated with an increasing hazard of death. Increasing CD4 counts and antiretroviral treatment during TB treatment were protective against death. In HIV-uninfected TB cases, advancing age was independently associated with death, whereas smear-positive disease was protective.

**Conclusion:**

We identified several independent predictors of death during TB treatment in resource-constrained settings. Our findings inform resource-constrained settings about certain subgroups of TB patients that should be targeted to improve mortality during TB treatment.

## Background

Over the past two decades, HIV co-infection has emerged as the greatest risk factor for developing active TB and causing TB-related deaths [1]. Reducing TB-related deaths is a priority in high burden countries. One of several strategies to reduce TB mortality is to identify those at increased risk of death. This strategy is especially important in developing countries where incident TB is frequent, and access to resources is constrained.

In regions of high TB incidence and high HIV prevalence, risk factors for TB case fatality include: older age (mostly >35 years) [2–5]; HIV positivity [2, 4, 6–9]; features of advanced HIV [8, 10–12]; smear-negative disease [3, 7, 9, 10, 13]; and malnutrition [4, 8, 14]. Studies in South Africa have determined predictors of TB mortality [6, 7, 15, 16]. However, these studies were of small or moderate sample size [6, 7, 15, 16], performed in gold mines with concomitant silicosis [6] or preceded the expansion of HIV services, trimethoprim-sulfamethoxazole chemoprophylaxis and antiretroviral treatment [6, 7, 15, 16].

Identifying predictors for mortality in South Africa in the ART era using TB surveillance data could inform resource allocation and strategies to reduce TB-related mortality. Here, we report our analyses of a complete electronic TB register of over 16,000 TB cases, where we determined predictors of death during TB treatment in a high HIV prevalence setting.

## Results

### Description of TB cases

Over a 36-month period, we recorded 17,735 TB cases. After exclusion of TB cases aged less than 16 years, we recorded 16,209 TB cases for 15,556 individuals. The median age was 33 years (IQR 26–41) and 48 % of TB cases were female (Table 1). Of those with known HIV status (n = 14,392), 72 % (n = 10,379) were infected with HIV and 28 % (n = 4013) were not. Of those infected with HIV, the median CD4 count was 146 cells/μL (IQR 65–266), 95 % received TMP-SMX chemoprophylaxis and 29 % received ART during TB treatment. Median duration of follow-up for all TB cases was 168 days (IQR 163–209). During TB treatment, 5.3 % and 10.5 % of TB cases died or were lost to follow-up, respectively. At least 76 % of TB cases were alive at the end of TB treatment.

**Table 1:** Description of TB cases by HIV status

	HIV uninfected (n = 4013)	HIV infected (n = 10,379)	HIV status not recorded (n = 1817)	Total (n = 16,209)
Sex^a^				
Data available	4013 (100.00)	10,379 (100.00)	1817 (100.00)	16,209 (100.00)
Sex female	1380 (34.39)	5798 (55.86)	639 (35.17)	7817 (48.23)
Age				
Data available	4012 (99.98)	10,378 (99.99)	1817 (100.00)	16,207 (99.99)
Median age (IQR)	32 (23–46)	33 (28–40)	31 (24–43)	33 (26–41)
<25	1380 (34.4)	1587 (15.29)	578 (31.81)	3545 (21.87)
25–35	907 (22.61)	4636 (44.67)	538 (29.61)	6081 (37.52)
35–45	690 (17.2)	2900 (27.94)	333 (18.33)	3923 (24.21)
45–60	764 (19.04)	1164 (11.22)	278 (15.3)	2206 (13.61)
>60	271 (6.75)	91 (0.88)	90 (4.95)	452 (2.79)
Anatomic site of TB				
Data available	4013 (100.00)	10,379 (100.00)	1817 (100.00)	16,209 (100.00)
Pulmonary	3358 (83.68)	6805 (65.57)	1465 (80.63)	11,628 (71.74)
Extra-pulmonary	543 (13.53)	2726 (26.26)	292 (16.07)	3561 (21.97)
Both	112 (2.79)	848 (8.17)	60 (3.30)	1020 (6.29)
Duration of TB treatment				
Data available	4013 (100.00)	10,379 (100.00)	1817 (100.00)	16,209 (100.00)
Median duration (IQR)	168 (164–194)	168 (161–219)	167 (161–187)	168 (163–209)
Retreatment for TB				
Data available	4013 (100.00)	10,379 (100.00)	1817 (100.00)	16,209 (100.00)
Retreated case	1001 (24.94)	3332 (32.10)	469 (25.81)	4802 (29.63)
TB smear results				
Data available	4013 (100.00)	10,379 (100.00)	1817 (100.00)	16,209 (100.00)
Smear negative	972 (24.22)	4773 (45.99)	528 (29.06)	6273 (38.70)
Smear positive	2716 (67.68)	4032 (38.85)	1103 (60.70)	7851 (48.44)
No smear performed	325 (8.10)	1574 (15.17)	186 (10.24)	2085 (12.86)
Outcome				
Data available	4013 (100.00)	10,379 (100.00)	1817 (100.00)	16,209 (100.00)
Alive	3237 (80.66)	7813 (75.28)	1333 (73.36)	12,383 (76.40)
Dead	132 (3.29)	641 (6.18)	78 (4.29)	851 (5.25)
Lost to follow-up	405 (10.09)	1079 (10.40)	210 (11.56)	1694 (10.45)
Transferred out	137 (3.41)	493 (4.75)	124 (6.82)	754 (4.65)
Not known	102 (2.54)	353 (3.40)	72 (3.96)	527 (3.25)
CD4 count				
Data available	–	6433 (61.98)	–	–
Median CD4 count (IQR)	–	146 (65–266)	–	–
TMP-SMX during TB treatment				
Data available	–	10,012 (96.46)	–	–
TMP-SMX during TB treatment	–	9520 (95.09)	–	–
ART during TB treatment				
Data available	–	8942 (86.15)	–	–
ART during TB treatment	–	2614 (29.23)	–	–

Data shows number of cases (n) and percentage (%) unless otherwise specified

*IQR* inter-quartile range, *TB* active tuberculosis, *TMP-SMX* trimethoprim sulfamethoxazole 160/80 mg chemoprophylaxis

^a^A total of 15,556 patients experienced 16,209 cases of tuberculosis. The female proportions for these 15,556 patients are: 48.6 % (7552/15,556) of all patients; 34.7 % (1347/3886) of HIV uninfected patients; 56.3 % (5584/9923) of HIV infected patients; and 35.6 % (621/1747) of patients with HIV status not recorded

### Predictors of death

**Fig. 1:**
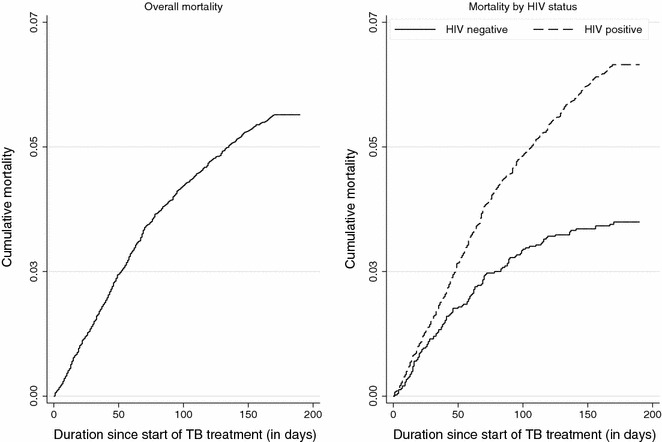
Kaplan Meier plot showing cumulative mortality during TB treatment: (1) overall mortality, and (2) mortality by HIV status

Cumulative mortality according to HIV status is shown in Fig. 1. Multivariate regression analyses of all TB cases found that independent predictors of death were increasing age, infection with HIV, a prior history of TB, the presence of extra-pulmonary TB and the presence of both pulmonary and extra-pulmonary TB (Table 2). Gender, smear result and the presence of only extra-pulmonary TB had no significant effect on mortality.

**Table 2:** Crude and adjusted hazard ratios with confidence intervals from a Cox model after multiple imputation for all deaths during TB treatment

	Crude		Adjusted		Adjusted (selected by AIC*)
	HR (95 % CI)	p value	HR (95 % CI)	p value	HR (95 % CI)
Sex female	1.05 (0.91–1.20)	0.51	1.08 (0.94–1.24)	0.28	
Age					
<25	Reference		Reference		Reference
25–35	1.88 (1.42–2.48)	0.00	1.53 (1.15–2.03)	0.00	1.52 (1.14–2.01)
35–45	2.37 (1.78–3.14)	0.00	1.93 (1.45–2.58)	0.00	1.90 (1.43–2.53)
45–60	3.54 (2.66–4.72)	0.00	3.25 (2.42–4.35)	0.00	3.20 (2.40–4.27)
>60	6.54 (4.65–9.19)	0.00	7.22 (5.11–10.22)	0.00	7.22 (5.11–10.19)
Anatomic site of TB					
Pulmonary	Reference		Reference		Reference
Extra-pulmonary	1.38 (1.18–1.62)	0.00	1.20 (0.99–1.45)	0.06	1.29 (1.10–1.51)
Both	1.62 (1.27–2.06)	0.00	1.45 (1.14–1.85)	0.00	1.46 (1.15–1.86)
Prior TB	1.28 (1.11–1.49)	0.00	1.16 (1.00–1.35)	0.05	1.16 (1.00–1.34)
HIV infected	1.82 (1.51–2.20)	0.00	1.94 (1.58–2.40)	0.00	2.00 (1.63–2.46)
TB smear result					
Smear negative	Reference		Reference		
Smear positive	0.73 (0.63–0.84)	0.00	0.93 (0.79–1.10)	0.41	
No smear performed	1.11 (0.91–1.36)	0.31	1.08 (0.87–1.34)	0.47	

*TB* tuberculosis, *ART* antiretroviral treatment, *TMP-SMX* trimethoprim sulfamethoxazole 160/80 mg chemoprophylaxis, *HR*  hazard ratio, *95* *% CI* 95 % confidence interval, *AIC* Akaike information criterion

Multivariate regression analyses of HIV-infected TB cases found that independent predictors of death were increasing age and female gender (Table 3). An increasing CD4 count and ART during TB treatment were protective against death. Smear result, history of prior TB, TMP-SMX prophylaxis during TB treatment and the presence of only extra-pulmonary TB had no clear effect on mortality.

**Table 3:** Crude and adjusted hazard ratios with confidence intervals from a Cox model after multiple imputation for all HIV-infected deaths during TB treatment

	Crude		Adjusted		Adjusted (selected by AIC*)
	HR (95 % CI)	p value	HR (95 % CI)	p value	HR (95 % CI)
Sex female	0.98 (0.85–1.14)	0.84	1.24 (1.06–1.45)	0.01	1.23 (1.06–1.44)
Age					
<25	Reference		Reference		Reference
25–35	1.25 (0.92–1.68)	0.15	1.24 (0.92–1.68)	0.17	1.25 (0.92–1.70)
35–45	1.58 (1.16–2.14)	0.00	1.60 (1.17–2.18)	0.00	1.62 (1.19–2.21)
45–60	2.39 (1.73–3.29)	0.00	2.50 (1.80–3.46)	0.00	2.54 (1.83–3.52)
>60	4.72 (2.99–7.44)	0.00	4.79 (3.00–7.65)	0.00	4.80 (3.00–7.66)
Anatomic site of TB					
Pulmonary	Reference		Reference		
Extra-pulmonary	1.16 (0.98–1.38)	0.09	1.14 (0.93–1.41)	0.21	
Both	1.44 (1.12–1.85)	0.01	1.29 (1.00–1.68)	0.05	
Prior TB	1.12 (0.95–1.32)	0.18	1.16 (0.98–1.37)	0.09	
TB smear result					
Smear negative	Reference		Reference		
Smear positive	0.98 (0.83–1.16)	0.83	1.07 (0.89–1.28)	0.47	
No smear performed	1.16 (0.93–1.45)	0.18	1.08 (0.85–1.36)	0.54	
CD4 count					
<50	Reference		Reference		Reference
50–100	0.79 (0.61–1.02)	0.07	0.79 (0.60–1.02)	0.07	0.78 (0.60–1.01)
100–200	0.55 (0.41–0.75)	0.00	0.54 (0.40–0.71)	0.00	0.53 (0.40–0.71)
200–350	0.47 (0.37–0.61)	0.00	0.43 (0.34–0.56)	0.00	0.43 (0.33–0.55)
350+	0.28 (0.18–0.43)	0.00	0.24 (0.16–0.37)	0.00	0.24 (0.16–0.37)
TMP-SMX chemoprophylaxis	1.01 (0.69–1.46)	0.96	0.88 (0.60–1.29)	0.52	
ART during TB treatment	0.76 (0.64–0.91)	0.00	0.60 (0.50–0.72)	0.00	0.61 (0.51–0.73)

*TB* tuberculosis, *ART* antiretroviral treatment, *TMP-SMX* trimethoprim sulfamethoxazole 160/80 mg chemoprophylaxis, *HR* hazard ratio, *95* *% CI* 95 % confidence interval, *AIC* Akaike information criterion

Multivariate regression analyses of HIV-uninfected TB cases found that increasing age was an independent predictor of death, whereas smear-positive disease was protective (Table 4). Gender, site of TB disease and history of prior TB were not associated with mortality.

**Table 4:** Crude and adjusted hazard ratios with confidence intervals from a Cox model after multiple imputation for all HIV-uninfected deaths during TB treatment

	Crude		Adjusted		Adjusted (selected by AIC*)
	HR (95 % CI)	p value	HR (95 % CI)	p value	HR (95 % CI)
Sex female	0.80 (0.56–1.15)	0.23	0.83 (0.58–1.19)	0.32	
Age, years					
<25	Reference		Reference		Reference
25–35	3.01 (1.35–6.68)	0.01	2.84 (1.28–6.30)	0.01	2.91 (1.31–6.45)
35–45	3.93 (1.73–8.96)	0.00	3.53 (1.55–8.05)	0.00	3.63 (1.59–8.26)
45–60	9.58 (4.56–20.11)	0.00	7.75 (3.65–16.42)	0.00	7.93 (3.74–16.79)
>60	20.38 (9.57–43.37)	0.00	16.14 (7.52–34.63)	0.00	16.24 (7.57–34.85)
Anatomic site of TB					
Pulmonary	Reference		Reference		
Extra-pulmonary	2.18 (1.48–3.21)	0.00	1.38 (0.85–2.22)	0.19	
Both	1.48 (0.60–3.66)	0.40	1.23 (0.49–3.06)	0.66	
Prior TB	1.94 (1.35–2.78)	0.00	1.41 (0.97–2.04)	0.07	1.39 (0.96–2.01)
TB smear result					
Smear negative	Reference		Reference		Reference
Smear positive	0.36 (0.25–0.52)	0.00	0.52 (0.35–0.78)	0.00	0.47 (0.33–0.68)
No smear performed	0.88 (0.50–1.54)	0.64	0.78 (0.43–1.40)	0.40	0.86 (0.49–1.51)

*TB* tuberculosis, *ART* antiretroviral treatment, *TMP-SMX* trimethoprim sulfamethoxazole 160/80 mg chemoprophylaxis, *HR* hazard ratio, *95* *% CI* 95 % confidence interval, *AIC* Akaike information criterion

Multivariate regression analyses stratified according to age found that certain risk factors differed according to HIV status (Tables 5, 6, 7). In younger patients (<45 years of age), HIV infection and the presence of both pulmonary and extra-pulmonary TB were associated with mortality (all TB patients, Table 5). In younger HIV infected patients, higher CD4 counts and initiation of ART during TB treatment were associated with reduced mortality (Table 6). In younger HIV uninfected patients, a positive smear result was protective against mortality (Table 7). In older patients (≥45 years), prior TB was associated with mortality (all TB patients, Table 5). In older HIV infected patients, female gender was associated with increased mortality whereas higher CD4 counts and initiation of ART during TB treatment were associated with reduced mortality (Table 6). Finally, in older HIV uninfected patients a positive smear result was protective against mortality (Table 7).

**Table 5:** Adjusted hazard ratios with confidence intervals from a Cox model after multiple imputation for all deaths during TB treatment according to age group (<45 years vs. ≥45 years of age)

	<45 years of age		≥45 years of age	
	aHR (95 % CI)	p value	aHR (95 % CI)	p value
Sex female	0.98 (0.83–1.16)	0.802	1.11 (0.86–1.44)	0.423
Anatomic site of TB				
Pulmonary	Reference		Reference	
Extra-pulmonary	1.16 (0.92–1.47)	0.213	1.20 (0.84–1.69)	0.313
Both	1.52 (1.14–2.01)	0.004	1.21 (0.72–2.05)	0.473
Prior TB	1.10 (0.92–1.33)	0.299	1.31 (1.01–1.70)	0.042
HIV infected	3.16 (2.34–4.27)	<0.001	1.18 (0.87–1.59)	0.282
TB smear result				
Smear negative	Reference		Reference	
Smear positive	0.99 (0.81–1.21)	0.904	0.76 (0.56–1.02)	0.068
No smear performed	1.21 (0.94–1.56)	0.142	0.87 (0.57–1.31)	0.501

*TB* tuberculosis, *ART* antiretroviral treatment, *TMP-SMX* trimethoprim sulfamethoxazole 160/80 mg chemoprophylaxis, *aHR* adjusted hazard ratio, *95* *% CI* 95 % confidence interval

**Table 6:** Adjusted hazard ratios with confidence intervals from a Cox model after multiple imputation for all HIV-infected deaths during TB treatment according to age group (<45 years vs. ≥45 years of age)

	<45 years		≥45 years	
	aHR (95 % CI)	p value	aHR (95 % CI)	p value
Sex female	1.10 (0.92–1.31)	0.310	1.44 (1.04–2.01)	0.030
Anatomic site of TB				
Pulmonary	Reference		Reference	
Extra-pulmonary	1.14 (0.89–1.46)	0.273	1.05 (0.66–1.66)	0.838
Both	1.33 (1.00–1.80)	0.052	1.09 (0.59 – 2.01)	0.775
Prior TB	1.13 (0.93–1.37)	0.234	1.39 (0.98–1.97)	0.066
TB smear result				
Smear negative	Reference		Reference	
Smear positive	1.08 (0.87–1.33)	0.471	0.94 (0.63–1.39)	0.741
No smear performed	1.16 (0.89–1.52)	0.262	0.81 (0.48–1.39)	0.447
CD4 count				
<50	Reference		Reference	
50–100	0.76 (0.57–1.02)	0.065	0.92 (0.54–1.59)	0.771
100–200	0.51 (0.37–0.70)	<0.001	0.62 (0.38–1.04)	0.068
200–350	0.44 (0.33–0.58)	<0.001	0.40 (0.23–0.70)	0.002
350+	0.23 (0.15–0.37)	<0.001	0.29 (0.13–0.67)	0.005
TMP-SMX chemoprophylaxis	0.83 (0.54–1.27)	0.393	0.96 (0.30–3.04)	0.936
ART during TB treatment	0.67 (0.44–0.83)	<0.001	0.44 (0.27–0.72)	0.001

*TB* tuberculosis, *ART* antiretroviral treatment, *TMP-SMX* trimethoprim sulfamethoxazole 160/80 mg chemoprophylaxis, *aHR* adjusted hazard ratio, *95* *% CI* 95 % confidence interval

**Table 7:** Adjusted hazard ratios with confidence intervals from a Cox model after multiple imputation for all HIV-uninfected deaths during TB treatment according to age group (<45 years vs. ≥45 years of age)

	<45 years		≥45 years	
	aHR (95 % CI)	p value	aHR (95 % CI)	p value
Sex female	0.84 (0.45–1.54)	0.565	0.82 (0.51–1.30)	0.391
Anatomic site of TB				
Pulmonary	Reference		Reference	
Extra-pulmonary	1.10 (0.45–2.67)	0.835	1.36 (0.76–2.44)	0.295
Both	1.37 (0.32–5.09)	0.671	1.22 (0.38–3.93)	0.744
Prior TB	1.64 (0.85–3.17)	0.139	1.33 (0.85–2.07)	0.206
TB smear result				
Smear negative	Reference		Reference	
Smear positive	0.41 (0.19–0.90)	0.027	0.57 (0.35–0.93)	0.025
No smear performed	1.02 (0.33–3.18)	0.970	0.87 (0.42–1.77)	0.697

*TB* tuberculosis, *ART* antiretroviral treatment, *TMP-SMX* trimethoprim sulfamethoxazole 160/80 mg chemoprophylaxis, *aHR* adjusted hazard ratio, *95* *% CI* 95 % confidence interval

## Discussion

In a surveillance population of over 16,000 adults with active TB, we found several easily identifiable and independent predictors of death during TB treatment. In those not infected with HIV, the risk of death increased with advancing age but decreased with smear-positive disease. In those infected with HIV, the risk of death increased with advancing age and female gender but decreased with a higher CD4 count and ART. These findings inform resource allocation strategies to reduce TB mortality in high HIV/TB settings.

Among HIV-infected adults receiving TB treatment, we found that women were at an increased hazard of death compared to men. This finding is interesting as HIV-infected men without TB typically have worse outcomes on ART, compared to women [27]. This discrepancy suggests that gender-TB or gender-ART interactions exist. Unmeasured confounding such as background mortality gender differences in this community could also explain this difference. Among HIV-uninfected persons with active TB, one study from Bolivia similarly found increased mortality among women, while other studies from Brazil, Italy, China, and Viet Nam found increased mortality among men [28–33].

Only 30 % of HIV-infected TB cases in our study received ART during TB treatment. This finding is likely because ART was only indicated for those with a CD4 count of less than 200 cells/μL, according to national guidelines at the time [18]. We found that ART reduced the risk of dying in our analysis. This finding occurred despite possible time-dependent confounding [34], wherein the observed benefit of ART would be smaller than anticipated because ART was started in sicker patients at various time-points during TB treatment. Our study adds impetus to current WHO guidelines that ART be initiated among all HIV-infected adults with active TB, regardless of CD4 count [35]. In those infected with HIV, interventions such as starting ART at higher CD4 counts may need to be prioritized in those of advanced age, or of the female gender. In those not infected with HIV, interventions will need to be prioritized in those with advancing age and those with smear-negative disease. Smear positive disease was protective of death among HIV-uninfected TB cases. Patients with smear negative disease may have had a diagnosis other than TB. Our previous study investigated reasons for clinical deterioration in patients on TB treatment. We found that 4 % of HIV infected TB patients and 9 % of HIV uninfected TB patients had alternate diagnoses to TB [36]. In HIV uninfected TB patients, these diagnoses included post-TB bronchiectasis, pulmonary silicosis, bacterial sepsis and lung cancer [37]. Smear negative disease could also reflect alternate illnesses such as sarcoidosis and lymphoma where inappropriate therapy could be fatal.

Among those infected with HIV, we found no association between smear results and death. Studies have found conflicting results with both smear positive and smear negative TB disease being associated with death [3, 9, 32]. TMP-SMX chemoprophylaxis has been shown to reduce mortality in HIV-infected patients [38, 39]. In our study, TMP-SMX chemoprophylaxis was not associated with reduced mortality. However, we were unable to assess the impact of this intervention on mortality because more than 95 % of our HIV-infected TB cases received TMP-SMX chemoprophylaxis.

Our study has several limitations. Loss to follow-up occurred in 10.5 % of cases and likely led to under-ascertainment of deaths [40]. Our study is retrospective, so our findings may be subject to random error, bias, and confounding. We minimized random error by utilizing a large surveillance population of over 16,000 TB cases. Also, trained health care personnel used a standardized data form during the study period, which likely reduced bias. Lastly, confounding was minimized by employing multivariate regression analyses including variables such as age, gender, HIV status, CD4 count if HIV-infected, TB smear result at TB diagnosis, history of prior TB, anatomic site of TB, and concurrent TMP-SMX and antiretroviral treatment during TB treatment. These variables have been shown to affect mortality during TB treatment [41]. We acknowledge that we did not have data on variables that may have influenced mortality, such as AIDS-defining illnesses; non-infective co-morbid illnesses; isoniazid prophylaxis therapy, nutritional factors; anemia; baseline performance status; socio-economic factors; smoking, alcohol and substance misuse; duration of symptoms prior to TB diagnosis; treatment adherence and drug-resistant strains; and HIV viral load [41]. We also were unable to correlate chest x-ray findings with mortality. Mortality during TB treatment may correlate with the extent of TB disease as evidenced by chest X-ray involvement and the presence or absence of cavity formation.

Despite these limitations, our study’s large sample size, standardized data collection and careful inputting of missing data add value to current literature. Our study identifies several easily recognizable factors that could be targeted to reduce TB mortality. Firstly, HIV testing should be performed for all TB patients. Secondly, we recommend a triple strategy among those infected with HIV. This triple strategy would include immediate (same day) CD4 counts at HIV diagnosis, expedited referral to HIV clinics for ART initiation and prioritization of ART for those of advancing age and of female gender. In HIV infected and HIV uninfected TB patients, our study identified those with advancing age as an independent risk factor for death. By identifying increasing age as a predictor of death, further research is now needed to determine whether excess TB deaths are due to age-related co-morbid illnesses or due to dysfunctional intracellular processes, such as autophagy [42]. Age related co-morbid illnesses such as ischemic heart disease, stroke, lower respiratory infections, diabetes and chronic obstructive pulmonary disease are listed in the top twenty causes of death in South Africa [43]. Importantly, a substantial proportion of TB patients have co-morbid illnesses such as hypertension (37 %) and diabetes (12 %) in this community [44]. If these co-morbid illnesses contribute to TB-related deaths, interventions would need to be implemented at TB diagnosis to diagnose and treat them.

## Conclusions

In a surveillance population of over 16,000 adults with active TB, several important predictors of death during TB treatment were recognized. In those not infected with HIV, the risk of death increased with advancing age but decreased with smear-positive disease. In those infected with HIV, the risk of death increased with advancing age and female gender but decreased with a higher CD4 count and ART. In a region of high TB incidence and high HIV prevalence with scarce resources, stratification at TB diagnosis by age, gender, HIV status and smear result would enable targeted interventions to reduce TB-related deaths.

## Methods

### Study population

Khayelitsha is located in Cape Town, South Africa and is a high density (>7500 inhabitants/km^2^) predominantly black African township consisting of more than 500,000 people [17]. From 2005 to 2007, annual TB case notification rates in Khayelitsha ranged from 1200 to 1400 per 100,000 people in the general population. TB patients in Khayelitsha are treated in 11 TB clinics administered by the City of Cape Town’s Health Department. According to national protocol, TB patients receive standardized TB treatment regimens using Directly Observed Therapy Short-course (DOTS) [18]. National guidelines at the time of our study recommended antiretroviral treatment (ART) for all TB patients with a CD4+ cell count less than 200 cells/μL or a history of a WHO stage 4 illness [18]. Extra-pulmonary TB—although a World Health Organization (WHO) stage 4 illness—was not an indication for ART unless the patient’s CD4+ count was less than 200 cells/μL. First-line ART during our study was stavudine, lamivudine, and either nevirapine or efavirenz. Efavirenz was preferred for adults receiving rifampin-based TB treatment. National guidelines also recommended daily trimethoprim-sulfamethoxazole (TMP-SMX, 160/800 mg) chemoprophylaxis [18]. Khayelitsha was one of the first townships in South Africa to integrate HIV and TB healthcare services. As a result, our previously described TB cohorts are characterized by high rates of voluntary counseling and testing of HIV status, as well as provision of TMP-SMX chemoprophylaxis (>95 %) [19–21].

Our study included all adult (>16 years of age) TB patients in Khayelitsha who initiated TB treatment from 1 January 2007 to 31 December 2009 and were recorded in ETR.net (Electronic Tuberculosis Register). ETR.net is an electronic database that was designed in consultation with TB managers in South Africa and developed with support from the US President’s Emergency Plan for AIDS Relief (PEPFAR) [22]. ETR.net is used for TB/HIV surveillance, program monitoring and evaluation [22]. It provides standardized cohort reports including case finding, smear results, outcomes of treatment and HIV testing and services for TB patients [22]. ETR.net has been implemented in several countries including South Africa, Guatemala, Mozambique, Namibia, Botswana, Swaziland and Tanzania [22].

### Study design

Variables obtained from the ETR.net database included age, gender, HIV status, TB smear result at TB diagnosis, history of prior TB, site of TB (pulmonary vs. extra-pulmonary vs. both), outcome (dead, alive, lost to follow-up or transferred to another health district) and duration of follow-up. Among those infected with HIV, variables such as CD4 count, ART use during TB treatment, and TMP-SMX use during TB treatment were also recorded. In this study, we included all TB patients receiving TB treatment at one of the 11 TB clinics in Khayelitsha. A TB death was defined according to the WHO criteria, which is ‘any death during TB treatment, irrespective of cause’ [23]. Patients lost to follow-up and transferred out were censored at the time of their last visit. The Human Research Ethics Committee of the University of Cape Town and the Cape Town City Health Department approved this study.

### Statistical methods

Patient characteristics (gender, age, TB site, TB history, TB smear, outcome) were summarized by HIV status using medians with interquartile ranges (IQR) or proportions. We also explored the effect of CD4 count, TMP-SMX chemoprophylaxis, and ART use on death in HIV-infected patients. Age and CD4 count were described as continuous variables and used as categorical variables in modeling. On the basis of the assumption that data were likely missing at random [24], we used multiple imputations by chained equations [25] to impute missing age [n = 1 (0.02 % of HIV uninfected data); n = 1 (0.01 % of HIV infected data)], CD4 [n = 3946 (38.02 % of HIV infected data)], TMP-SMX chemoprophylaxis [n = 367 (3.54 % of HIV infected data)], and ART data for HIV-infected patients [n = 1437 (13.85 % of HIV infected data)]. The multiple imputation models for the above variables included all measured variables as well as follow-up time. We used Stata’s implementation of the chained equation approach and used predictive mean matching to guarantee that imputed CD4 counts and age lie within the range of the observed data. All results of the statistical models relied on the imputed datasets and were combined by Rubin’s rules [24].

Cumulative mortality of patients was visualized using the Kaplan–Meier estimator. To identify risk factors for death, we used Cox proportional hazards models and reported hazard ratios and their respective 95 % confidence intervals. All patients on TB treatment were considered and included in the model. We performed sub-analyses for both HIV positive and HIV negative populations. We also performed model selection using Akaike’s Information Criterion to obtain the most predictors of death [26].
